# A Guideline of Cross Taping for the Assessment and Treatment of Musculoskeletal Disorder

**DOI:** 10.3390/healthcare9060717

**Published:** 2021-06-11

**Authors:** Jung-Hoon Lee

**Affiliations:** Department of Physical Therapy, College of Nursing, Healthcare Science and Human Ecology, Dong-Eui University, Busan 47340, Korea; dreampt@hanmail.net; Tel.: +82-51-890-4222

Recently, the use of cross taping has increased, especially among clinicians in Europe, in the treatment of various musculoskeletal disorders. Nevertheless, many clinicians may remain unfamiliar with the cross-taping method, while some scholars may regard it as an unscientific method [1]. Cross taping was first introduced by Danaka as a method involving a rectangular cross tape based on an equidistant cross of three and four polyester tapes with non-elastic adhesive acryl coating. The cross tape is applied to an acupuncture or muscle tone point in the treatment of various musculoskeletal disorders or general medical conditions [1,2]. In previous studies, cross taping was shown to reduce pre-menstrual pain [3], increase proprioception and balance in the neck and ankle areas [4], and reduce pain and promote functional improvement in the treatment of shoulder impingement syndrome, where cross tape was combined with kinesiology tape and applied to the levator scapulae muscle [5].

The modified self-O-ring test (SOT) is a modified version of the sensitive and non-invasive bi-digital O-Ring test, originally developed by Omura and Yoshiaki to obtain various pathological data [1,6]. The modified SOT in cross taping allows the subjective test of the level of O-ring shaped finger resistance to identify the point of cross taping, when the patient first uses the thumb and the middle finger to form an O-ring in contact with the little finger and then uses the index finger of the opposite hand to force open the ring [1,2,7,8]. A relatively high level of O-ring shaped finger resistance in the modified SOT is considered a positive response, and a relatively low level of resistance, a negative response [1,2]. The modified SOT for the point of pain or high muscle tone has been reported to show a positive response, indicating a relative strength increase in O-ring shaped finger resistance in comparison to that of the surrounding tissues [1,2,7].

Cross tape is a 3 × 4 rectangle, where the line of three that indicates as positive in oriental medicine and the line of four that indicates as negative are in an equidistant cross [2]. Prior to cross taping, the direction to apply the cross tape in should be tested at the point that showed a positive response in the modified SOT [1,2]. The direction of cross taping is divided into the left direction type (LDT) and the right direction type (RDT) [1,2]. The method of determining LDT or RDT is as follows: The cross tape is applied to a point of muscle fiber on the forearm by tilting the line of four to 45° on the left, followed immediately by the modified SOT at the point where the left clavicle meets the sternocleidomastoid muscle (LDT check point) [1]. The cross tape is then applied to a point of muscle fiber on the forearm by tilting the line of four to 45° on the right, followed immediately by the modified SOT at the point where the right clavicle meets the sternocleidomastoid muscle (RDT check point) [1]. If the modified SOT shows a higher level of O-ring shaped finger resistance when the line of four has been applied at a 45° tilt to the left, then the direction of cross taping is determined as the LDT [1,2,7].

Another method of determining the direction of cross taping is as follows: The diagnostician collects four fingers and sweeps up the forearm muscle fiber of the patient by 45° to the left and to the right and carries out the modified SOT immediately at the point of sweep to check which direction has given a stronger positive response [1]. If the modified SOT shows a stronger positive response for the 45° sweep to the right of the muscle fiber, then the direction of cross taping is determined as the RDT [1]. The test result should be identical regardless of the muscle type.

In a previous study, the modified SOT showed inconsistent results when the direction of cross taping varied, even if the point of application remained identical [7]. The maximal grip strength was shown to be higher when the cross taping was in the direction of the positive response in the modified SOT, and when Cohen’s Kappa for the modified SOT and the maximal grip strength was in perfect agreement [7]. Cross taping in the direction of the positive response in the modified SOT, compared to cross taping in the opposite direction, was reported to significantly reduce muscle tone and increase local blood circulation [9]. Due to a serious lack of studies to date on cross taping, from randomized controlled studies to meta-analyses to systematic reviews [10], most clinicians rely on the information provided by the manufacturer on the product website [11]. Thus, in practice, clinicians and researchers apply the cross tape to the myofascial trigger points, acupuncture points, and local pain areas or joints without considering the direction of application. In a previous study reporting a lack of change in the muscle tone after applying the cross tape to the upper trapezius trigger point, no test had been performed on the direction of application before cross taping [10]. In another study reporting a lack of significant effect in primary dysmenorrhea when cross taping was applied to the greater trochanter area, no test on the direction of application had been performed [12]. Thus, it is recommended that the cross taping be applied to a muscle area at a 45° tilt in the direction (LDT or RDT) of the positive response in the modified SOT.

For the cross taping at a joint junction, however, the application should involve the rectangular shape of the 3 × 4 or 4 × 3 cross tape type, in contrast to the left or right 45° tilt in the case of a muscular area [1,2]. For the cross taping at a nerve entrapment point, likewise, the application should involve the rectangular shape of the 3 × 4 or 4 × 3 cross tape type.

The rectangular 3 × 4 or 4 × 3 cross tape type may also be applied in the case of a surgical scar [1]. The skin of surgical scars shows a greatly reduced elasticity compared to normal skin as it is in a restored state after the destruction of the continuity from the skin to the muscle tissue. The muscle tissue beneath the skin is thus under the influence of reduced elasticity [13]. The load on the muscle in daily activities is dispersed among various parts of the body through stretched skin to reduce the burden on individual muscle tissues, which reduces muscle fatigue [13]. However, with reduced elasticity, the skin of surgical scars is unable to efficiently disperse the load during activity, and certain muscle tissues end up receiving the entire load, inevitably increasing the muscle tone [13]. Thus, the application of the rectangular 3 × 4 or 4 × 3 cross tape type on a surgical scar is likely to reduce the skin and muscle tone and consequently allow easier movements [1].

In the absence of a positive response in the modified SOT after cross taping, the position or angle of the cross tape should be slightly modified, and the cross tape should be applied after another modified SOT since the direction of muscle fiber may vary in each patient [1]. It is also recommended that the modified SOT be carried out on each day of applying the cross tape, even in a single patient, as the direction of application may vary according to the daily state of the patient [1].

Prior to cross taping, the skin of the patient should be dry and clean without any lotion or other ointments [1]. The maximum period of cross taping should not exceed 24 h because, first, the patient’s various daily or sports activities may cause sweating and consequent skin irritation [1] and, second, the point and direction of cross taping may be altered due to a change in the state of the patient [1,2]. In the case of itching, redness, edema, hives or headache after cross taping, the cross tape should be immediately removed regardless of the duration of the application [1,2].

While the use of cross tape by clinicians has gradually increased, cross taping has so far been applied without the modified SOT before and after application. Thus, following the guideline presented in this study upon cross taping is anticipated to contribute to the treatment of patients, as well as to the studies on the effects of cross taping.

## Data Availability

Not applicable.
